# Adhesion States Greatly Affect Cellular Susceptibility to Graphene Oxide: Therapeutic Implications for Cancer Metastasis

**DOI:** 10.3390/ijms25031927

**Published:** 2024-02-05

**Authors:** Keiko Morotomi-Yano, Shinya Hayami, Ken-ichi Yano

**Affiliations:** 1 Institute of Industrial Nanomaterials, Kumamoto University, 2-39-1 Kurokami, Chuo-ku, Kumamoto 860-8555, Japan; 2 Department of Chemistry, Faculty of Advanced Science and Technology, Kumamoto University, 2-39-1 Kurokami, Chuo-ku, Kumamoto 860-8555, Japan

**Keywords:** graphene oxide, biocompatibility, cell adhesion, autophagy, metastasis

## Abstract

Graphene oxide (GO) has received increasing attention in the life sciences because of its potential for various applications. Although GO is generally considered biocompatible, it can negatively impact cell physiology under some circumstances. Here, we demonstrate that the cytotoxicity of GO greatly varies depending on the cell adhesion states. Human HCT-116 cells in a non-adhered state were more susceptible to GO than those in an adherent state. Apoptosis was partially induced by GO in both adhered and non-adhered cells to a similar extent, suggesting that apoptosis induction does not account for the selective effects of GO on non-adhered cells. GO treatment rapidly decreased intracellular ATP levels in non-adhered cells but not in adhered ones, suggesting ATP depletion as the primary cause of GO-induced cell death. Concurrently, autophagy induction, a cellular response for energy homeostasis, was more evident in non-adhered cells than in adhered cells. Collectively, our observations provide novel insights into GO’s action with regard to cell adhesion states. Because the elimination of non-adhered cells is important in preventing cancer metastasis, the selective detrimental effects of GO on non-adhered cells suggest its therapeutic potential for use in cancer metastasis.

## 1. Introduction

Graphene oxide (GO) is an oxidized form of graphene that is a two-dimensional nanosheet with a single layer of sp^2^-bonded carbon [1,2]. GO has received particular interest in the life sciences because of its unique properties, such as a high hydrophilicity and consequent dispersibility in aqueous solutions, a surface functionalization ability owing to oxygen-based functional groups, and strong interactions with biological macromolecules [3]. Thus, GO is now considered promising for various biological and medical applications [4,5]. First, GO offers good prospects for the repair and regeneration of various human organs, including bones, cartilage, skin, muscles, and nerves [6,7,8,9,10]. Second, GO has great potential as a vehicle for the delivery of macromolecules, such as drugs and genes [5,11]. Moreover, GO can be used against microbial and fungal growth and viral infection [12,13,14,15]. Furthermore, GO has raised considerable interest in the development of novel GO-based biosensors for clinical diagnosis [16,17].

To assess the safety and risk of GO in the human body, much effort has been devoted to deciphering how GO acts on human cells. Many studies employing cultured human cell lines have demonstrated that GO is generally biocompatible [18,19,20] but exhibits cytotoxicity under certain circumstances, such as at high GO concentrations [18,21]. Several distinct mechanisms for GO cytotoxicity have been proposed so far, and they appear to act on cells cumulatively. First, GO attaches to the cell surface and causes physical damage to the cell membrane [22,23,24]. GO also preferentially associates with specific species of membrane lipids, leading to alterations in the lipid composition of the cell membrane [25,26,27]. A portion of the membrane-attached GO enters cells via either a passive [28,29] or a biologically active [30,31,32] mechanism and eventually causes intracellular responses, such as oxidative stress [33,34].

The most prominent consequence of GO-induced cellular damage is apoptosis [35]. Apoptosis is energy-dependent programmed cell death, and its execution requires sufficient cellular ATP [36,37,38]. In addition to apoptosis, GO treatment frequently induces autophagy, which is a mechanism for the degradation of various cellular components [31,39]. Autophagy can be induced via starvation and other cellular stress and serves to maintain energy homeostasis [40]. Notably, the presence of fetal bovine serum (FBS) significantly reduces the cytotoxicity of GO [23,41]. FBS contains serum proteins at high concentrations. Because GO adsorbs various proteins [42,43], its surface is readily coated with serum proteins in FBS-containing medium, leading to the suppression of GO cytotoxicity. The mitigation of the detrimental GO effects by FBS strongly suggests that GO cytotoxicity is primarily attributed to the direct interaction between GO and the cell surface [41].

To date, many studies on GO cytotoxicity have utilized human cell lines derived from solid tumors, such as A549 [18,21], HeLa [29], and HCT-116 [31]. Under cultured conditions, these cells generally adhere to, and spread on the bottom of, a culture dish, and they are thus collectively called adherent cells. For most adherent cells, adhesion to an extracellular scaffold is essential for their survival and proliferation, which is known as anchorage dependence [44]. The loss of adhesion leads to the induction of apoptotic cell death, which is referred to as anoikis [44]. In the human body, anoikis eliminates non-adhered malignant cells liberated from their primary sites and thus serves as a defense mechanism against cancer metastasis [45].

Despite the importance of adhesion in cellular physiology and cancer metastasis, little attention has been paid to the relationship between adhesion states and GO effects, which prompted us to compare the responses of adhered and non-adhered cells to GO. In this study, we found that adhesion states profoundly affect the cellular susceptibility to GO: the effects of GO on cell viability, intracellular ATP levels, and autophagy were more evident in non-adhered cells than adhered ones. Our observations provide a novel insight into the mechanism of GO action and also have possible implications for a new GO-based approach against cancer metastasis.

## 2. Results

### 2.1. Increased Susceptibility of Non-Adhered Cells to GO

**Figure 1:**
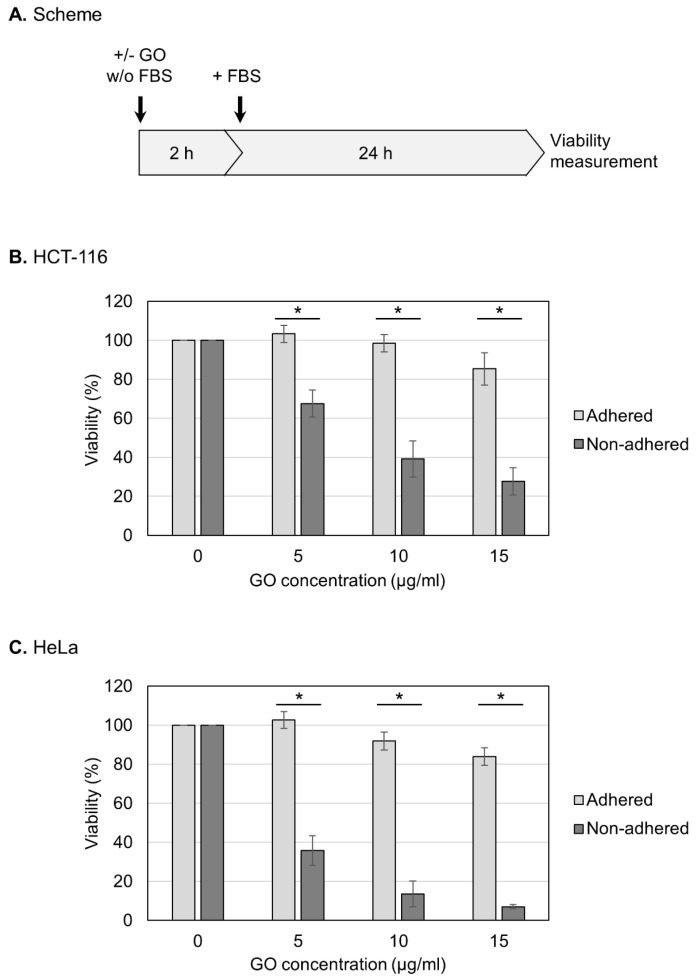
Increased susceptibility of non-adhered cells to GO. (**A**) Experimental scheme for viability assays. Cells were treated with GO in a medium without FBS. After 2 h GO treatment, FBS was added to the culture. Cell viability was measured at 24 h after FBS addition. (**B**) The effects of GO on the viabilities of adhered and non-adhered HCT-116 cells. HCT-116 cells in either adhered or non-adhered states were incubated with GO at 0, 5, 10, and 15 µg/mL for 2 h. After the addition of FBS, the cell culture was continued for 24 h, and the cell viability was measured. Average values with SD were calculated from six independent experiments. *: statistically significant; *p*-values are 3.2 × 10^−06^ (5 µg/mL), 1.7 × 10^−06^ (10 µg/mL), and 1.7 × 10^−07^ (15 µg/mL). (**C**) The effects of GO on the viabilities of adhered and non-adhered HeLa cells. GO treatment and viability measurement were performed on HeLa cells as described in B. Average values with SD were calculated from six independent experiments. *: statistically significant; *p*-values are 7.5 × 10^−08^ (5 µg/mL), 2.1 × 10^−09^ (10 µg/mL), and 4.0 × 10^−08^ (15 µg/mL).

In this study, we used GO that had been characterized in detail in our previous study [46]. Briefly, GO was suspended in pure water, dispersed via sonication, and subjected to characterization. GO was estimated to exist as rectangular monolayer sheets that were approximately 1 nm thick and 2–4 µm long [46]. The oxygen degree was measured as 33%, and most of the oxygen functional groups were epoxy groups [46]. Before treating cells, aqueous dispersed GO was adjusted to a neutral pH and diluted in a culture medium that lacked FBS. GO was then reacted with HCT-116 cells, which are relatively resistant to anoikis and can survive under non-adhered conditions for prolonged periods [47,48,49]. For the measurement of viability, HCT-116 cells in either an adherent or non-adhered state were treated with GO in the absence of FBS for 2 h (Figure 1A). After the addition of FBS, the cell culture was further continued, and the cell viability was measured at 24 h (Figure 1A).

First, we examined the effects of GO on the viability of human HCT-116 cells in an adherent state. As shown in Figure 1B, we observed that the adhered cells were relatively resistant to GO: the cell viabilities after 2 h treatment with 5 and 10 μg/mL GO were indistinguishable from untreated samples, and 15 μg/mL GO caused a slight decrease in the viability. We next repeated the experiments using cells in a non-adhered state and found that GO was highly detrimental to non-adhered cells compared to adhered ones; even at 5 μg/mL, GO treatment caused a significant reduction in the viability (Figure 1B). We performed the same experiments using HeLa cells and obtained similar results: non-adhered HeLa cells were more sensitive to GO than adhered ones (Figure 1C).

**Figure 2:**
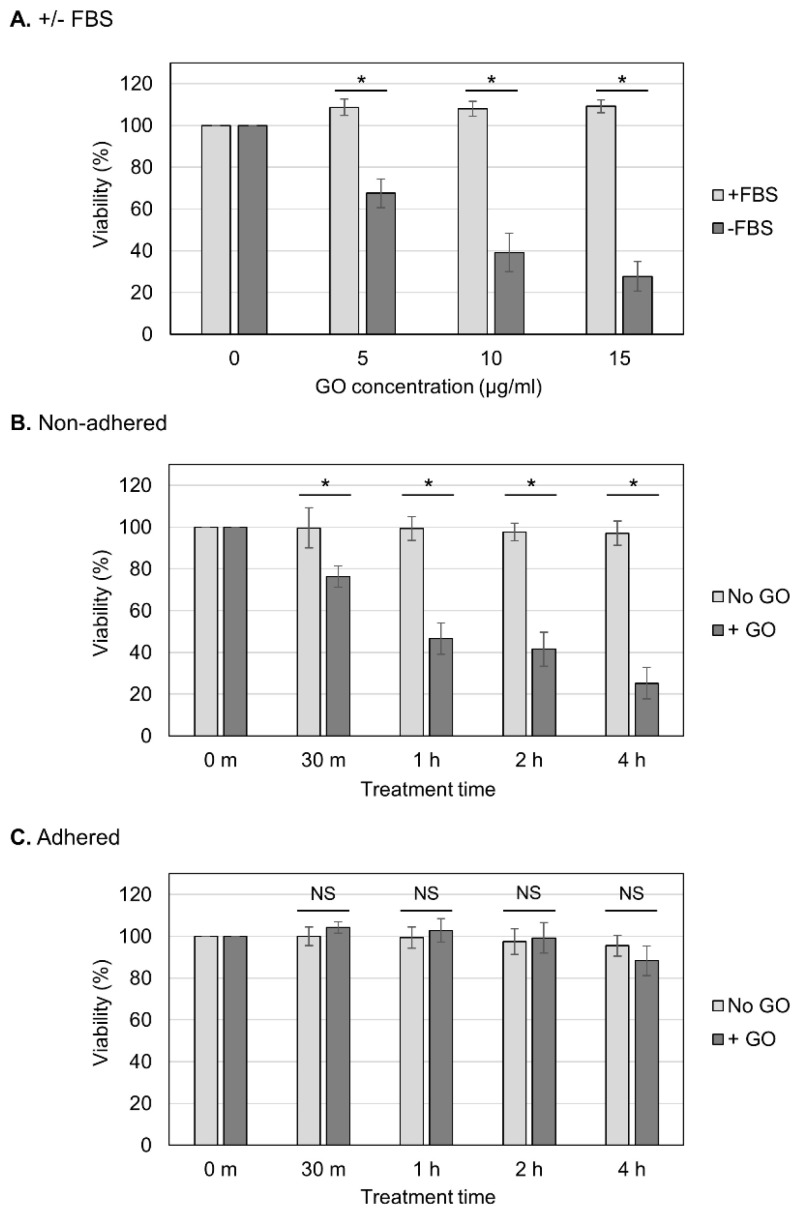
Effects of different periods of GO treatment on cell viability. (**A**) The neutralization of the detrimental effect of GO via FBS. Non-adhered HCT-116 cells were treated with GO in the presence of FBS, and their viabilities were measured, as shown in Figure 1B (+FBS, light-gray bars). The data for GO treatment without FBS are the same as those in Figure 1B (−FBS, dark-gray bars). Average values with SD were calculated from six independent experiments. *: statistically significant; *p*-values are 1.6 × 10^−06^ (5 µg/mL), 1.2 × 10^−06^ (10 µg/mL), and 4.9 × 10^−08^ (15 µg/mL). (**B**) The effects of different periods of GO treatment on non-adhered cells. Non-adhered HCT-116 cells were treated with 10 µg/mL GO without FBS. In parallel, cells were incubated without GO or FBS. After the indicated periods of incubation, FBS was added to the cultures. Cells were further incubated for 24 h, and the cell viability was measured. Average values with SD were calculated from six independent experiments. *: statistically significant; *p*-values are 4.0 × 10^−04^ (30 m), 1.4 × 10^−07^ (1 h), 8.6 × 10^−07^ (2 h), and 1.0 × 10^−08^ (4 h). (**C**) The effects of different periods of GO treatment on adhered cells. Experiments were performed on adhered HCT-116 cells, as described in B. Average values with SD were calculated from six independent experiments. NS: not statistically significant; *p*-values are 0.09 (30 m), 0.37 (1 h), 0.54 (2 h), and 0.11 (4 h).

We aimed to determine whether the observed GO toxicity to non-adhered cells could be attenuated via FBS (Figure 2A). We observed that the presence of FBS during GO treatment diminished the detrimental effect of GO, which is consistent with the previous finding that FBS mitigates GO cytotoxicity [23,41]. We next analyzed the effects of different periods of GO treatment (Figure 2B,C). Cells were treated with GO from 30 min to 4 h, and, subsequently, FBS was added to the cultures to neutralize the effects of GO (Figure 2B,C). We observed that longer GO treatment caused a greater reduction in the viability of non-adhered cells (Figure 2B). On adhered cells, prolonged GO treatment did not have significant effects on the viability (Figure 2C). These results confirmed the striking difference in cellular susceptibility to GO between non-adhered and adherent states.

### 2.2. Partial Induction of Apoptosis via GO in Non-Adhered Cells

**Figure 3:**
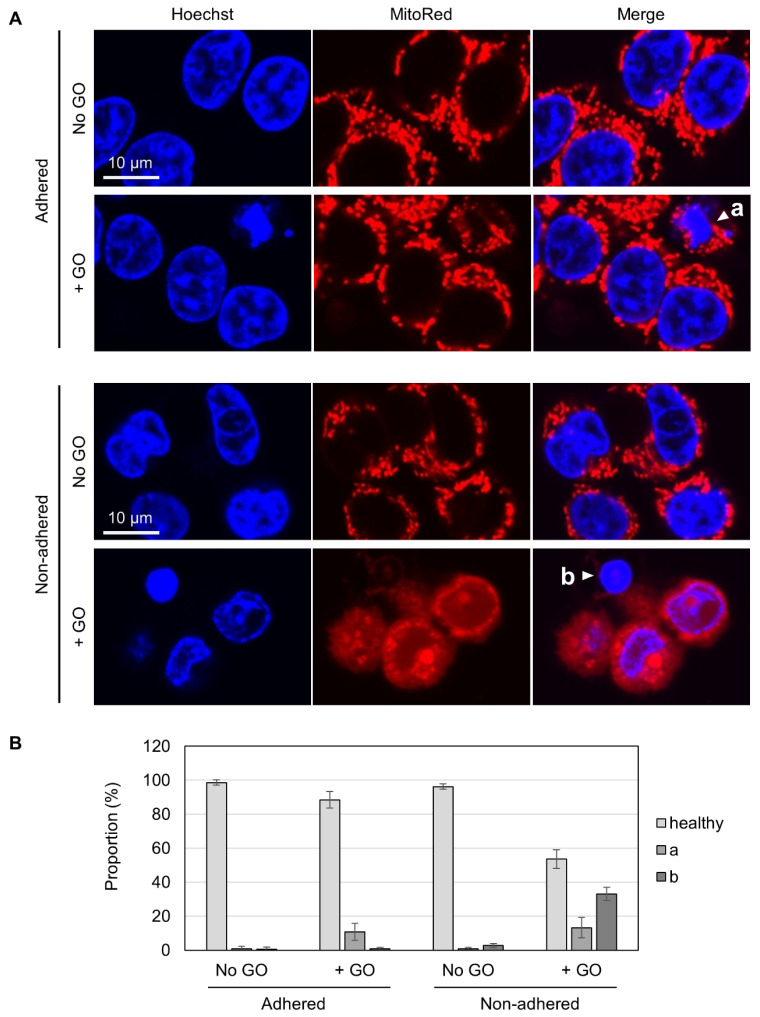
Fluorescence microscopy of GO-treated cells. (**A**) HCT-116 cells in either non-adhered or adhered states were treated with or without 10 µg/mL GO in the absence of FBS for 2 h. After the addition of FBS, cells were incubated for 4 h and subsequently stained with Hoechst 33342 and MitoRed. White arrowheads (a and b) indicate a cell with a condensed nucleus and normal mitochondrial signals (a) and a cell with a condensed nucleus and reduced mitochondrial signals (b), respectively. (**B**) The proportions of cells with condensed nuclei and reduced mitochondrial signals. Cells were microscopically inspected and classified into three categories (healthy, a: a condensed nucleus and normal mitochondrial signals, b: a cell with a condensed nucleus and reduced mitochondrial signals). Average values with SD were calculated from six independent experiments.

To gain insights into the differential GO effects depending on cellular adhesion states, we carried out fluorescence microscopy. Living cells were costained with Hoechst 33342 for nuclear DNA and MitoRed for active mitochondria. Without GO treatment, almost all cells appeared healthy, irrespective of adhesion states, as judged via both blue nuclear staining and red punctate staining for mitochondria (Figure 3A). In GO-treated adhered cells, a small fraction of cells exhibited nuclear condensation (marked as “a” in Figure 3A), which is known to be a characteristic feature of apoptotic cells [50]. Most of these apoptotic cells had punctate red staining, suggesting that mitochondrial activity was maintained, at least to some extent, in these cells. Among GO-treated non-adhered cells, on the other hand, we found cells with condensed nuclei and very faint mitochondrial staining (marked as “b” in Figure 3A). Because the MitoRed dye stains functionally intact mitochondria, the disappearance of punctate red staining suggested that the mitochondrial function was severely compromised. We quantified the numbers of these cell types and found that a substantial fraction of GO-treated non-adhered cells had nuclear condensation and compromised mitochondria (shown as “b” in Figure 3B).

**Figure 4:**
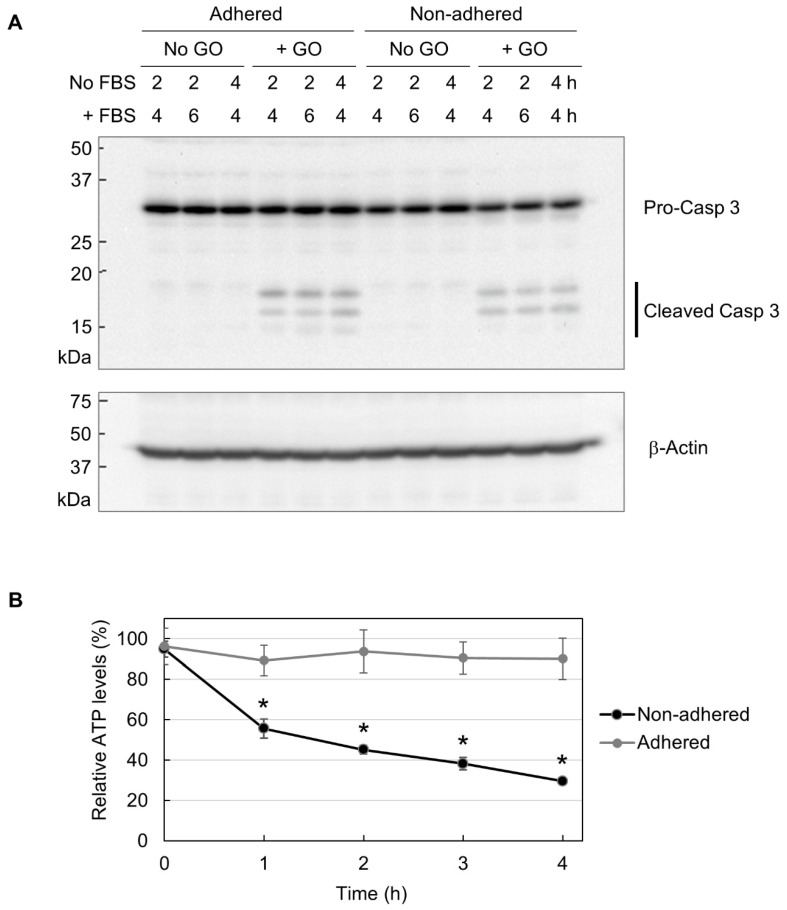
The partial induction of apoptosis due to decreased cellular ATP. (**A**) The Western blot analysis of caspase 3. Adhered and non-adhered HCT-116 cells were treated with 10 µg/mL GO for 2 or 4 h, as indicated. FBS was added to the cultures, and incubation was continued for the indicated periods. Cells were subsequently collected and subjected to Western blot analysis of caspase 3. The positions of the precursor and the cleaved forms of caspase 3 are indicated (**upper panel**). The membrane was reprobed with β-actin antibody (**lower panel**). (**B**) The ATP levels of GO-treated HCT-116 cells in adhered and non-adhered states. Adhered and non-adhered HCT-116 cells were incubated with or without 10 µg/mL GO for the indicated periods in the absence of FBS. The ATP levels of GO-treated samples relative to untreated ones were obtained. Average values with SD were calculated from six independent experiments. *: statistically significant; *p*-values are 1.2 × 10^−05^ (1 h), 6.6 × 10^−05^ (2 h), 2.6 × 10^−06^ (3 h), and 2.3 × 10^−05^ (4 h).

Next, we performed Western blot analysis of caspase 3, which is located downstream of the apoptotic pathway and plays a crucial role in apoptosis execution [51,52]. Caspase 3 exists as an inert precursor and is activated via partial proteolysis during apoptosis induction [51,52]. Cleaved caspase 3 is widely regarded as a molecular marker for apoptosis induction. As shown in Figure 4A, GO treatment resulted in caspase 3 cleavage in both adhered and non-adhered cells to a similar extent. Because GO was more detrimental to non-adhered cells than adhered ones, the results of Western blotting mean that apoptosis induction does not account for the observed difference in GO susceptibility between adhered and non-adhered cells.

Apoptosis is known as an energy-driven process and thus requires a sufficient level of cellular ATP [36,37,38]. As assessed via MitoRed staining, as shown in Figure 3, GO treatment caused the impairment of mitochondrial functionality in non-adhered cells. Thus, we next investigated whether GO differentially affects cellular ATP levels depending on adhesion states. We treated adhered and non-adhered cells with GO and measured their ATP levels over time. We observed that GO treatment led to a rapid ATP decrease in non-adhered cells, while the ATP levels in adhered cells were maintained during GO treatment (Figure 4B). Together with the results of the microscopy of MitoRed-stained cells (Figure 3) and Western blot analysis of caspase 3 (Figure 4A), we speculated that GO treatment initiates apoptosis in non-adhered cells, but a simultaneous decrease in intracellular ATP interferes with the proper execution of apoptosis, leading to cell death with partial signs of apoptosis.

### 2.3. Increased Autophagy in GO-Treated Non-Adhered Cells

**Figure 5:**
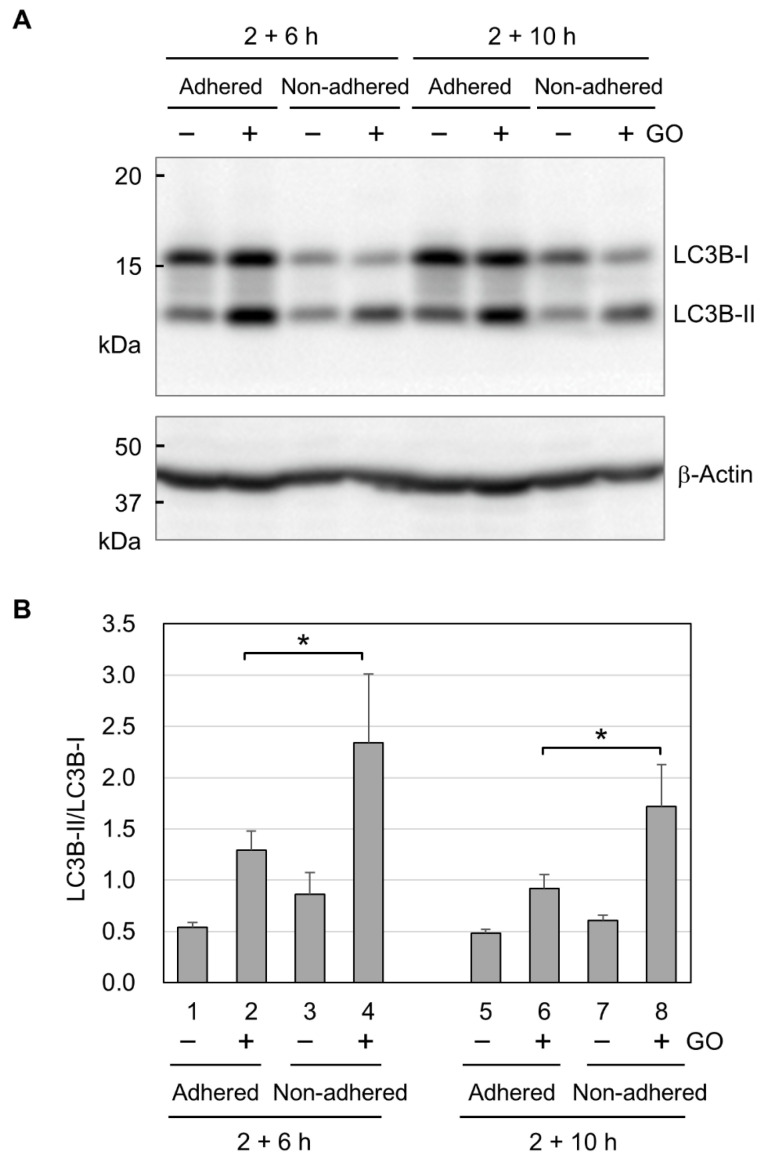
The activation of autophagy by GO. (**A**) Representative image of the Western blot analysis of LC3B. Adhered and non-adhered HCT-116 cells were incubated with or without 10 µg/mL GO for 2 h. After FBS addition, incubation was further continued for 6 or 10 h, as indicated. Cells were subsequently subjected to the Western blot analysis of LC3B. The positions of the non-lipidated (LC3B-I) and lipidated (LC3B-II) forms of LC3B are shown. (**B**) LC3B-II/LC3B-I ratios. Western blot signals for LC3B-I and LC3B-II were quantified, and LC3B-II/LC3B-I ratios were obtained. Average values with SD were calculated from four independent experiments. *: statistically significant; *p*-values are 0.047 (2 + 6 h) and 0.040 (2 + 10 h).

Finally, we analyzed autophagy activation by GO in adhered and non-adhered cells. We performed Western blot analysis for two forms of LC3B. LC3B-I exists in the cytosol and is modified with lipidation to become LC3B-II during autophagy [53]. Because the conversion of LC3B-I to LC3B-II shows a good correlation with autophagosome formation [54], the LC3B-II/LC3B-I ratio is widely used as a molecular marker for autophagy. In Western blotting, we detected increased signals for LC3B-II in both adhered and non-adhered cells (Figure 5A), indicating that GO activates autophagy in adhered and non-adhered cells. We next quantified the Western blot signals for LC3B-I and LC3B-II and calculated the LC3B-II/LC3B-I ratios. As shown in Figure 5B, the LC3B-II/LC3B-I ratios in GO-treated non-adhered cells were higher than those in GO-treated adhered cells. This result suggests that GO treatment induces a stronger activation of autophagy in non-adhered cells than adhered ones.

## 3. Discussion

GO holds great promise for various applications in the life sciences, and a comprehensive understanding of GO action on human cells is important for safe and effective GO usage in the human body. In this study, we demonstrated that cell adhesion states profoundly affect cellular susceptibility to GO. Non-adhered cells were more sensitive to GO and underwent cell death with partial signs of apoptosis. GO treatment caused a marked decrease in intracellular ATP levels and augmentation of autophagy in non-adhered cells. These observations reveal a novel aspect of GO action on human cells and contribute to a better understanding of the mechanism underlying the cytotoxicity of GO. We used monolayered rectangular GO with 33% oxygen in this study, and the size and oxygen content may influence the differential GO effects depending on the adhesion state, which merits further investigation.

Although we observed the marked cytotoxicity of GO in non-adhered cells, apoptosis induction by GO was similar between adhered and non-adhered cells, as assessed by the formation of cleaved caspase 3 (Figure 4A). This means that the difference in apoptosis induction does not account for the observed difference in GO cytotoxicity depending on adhesion states. As shown in Figure 4B, GO treatment markedly decreased intracellular ATP levels in non-adhered cells, but those in adhered ones were largely unaffected. In addition, autophagy was more evident in non-adhered cells than in adhered ones (Figure 5B). Based on these observations, we speculate that GO treatment initiates apoptosis, but a rapid ATP reduction interferes with the proper execution of apoptosis. Apoptosis is an energy-dependent process and requires a sufficient level of cellular ATP [36,37,38]. A previous study has shown that, after the induction of apoptosis, normal levels of intracellular ATP are maintained in the initial stage of apoptosis for several hours [38]. When ATP production is pharmacologically inhibited, an apoptosis-inducing stimulus causes non-apoptotic cell death [37]. For these reasons, we infer that GO treatment initiates the apoptotic process in non-adhered cells but eventually results in non-apoptotic death, primarily due to ATP depletion. This idea should be validated through more detailed analysis in future research.

This study shows a novel link between GO and cell adhesion states, which raises the next question of why cell detachment causes a higher cellular susceptibility to GO. Many studies on GO cytotoxicity have shown the importance of the interaction of GO with the cell surface, and we speculate that adhesion states may affect the interaction between GO and the cell surface. One of the major differences between adhered and non-adhered cells is the surface area. A non-adhered cell alters its shape from flat to round and has a smaller cell surface area than an adhered one. The decrease in surface area may change the tension of the cell membrane if the cell volume is the same. Thus, one plausible mechanism is that the cell membrane of a spherical cell is prone to physical damage or lipid extraction by GO, which manifests as increased GO susceptibility in a non-adhered state. Another possibility is that GO cytotoxicity correlates with cytoskeletal organization, which differs between the adhered and non-adhered states. It would be intriguing to test whether the pharmacological stabilization/destabilization of cytoskeletons affects GO cytotoxicity in different adhesion states. Further research to test these possibilities will advance our understanding of how GO differentially affects human cells depending on adhesion states.

From the viewpoint of cancer medicine, our findings have important implications for novel therapeutic approaches against metastasis. Metastasis is the leading cause of death in cancer patients, and its prevention is critical for better outcomes in cancer therapy [55]. In the course of metastasis, malignant cells dissociate from their primary site and migrate to give rise to metastatic tumors at distant sites. The sensitization of non-adhered cells to cell death, such as anoikis, is recently regarded as an effective therapeutic strategy for metastasis [56,57]. This study demonstrated that GO preferentially exerts its detrimental effects on non-adhered cells. Because the selective elimination of dissociated cells could be beneficial in suppressing metastasis, our findings suggest the therapeutic potential of GO for metastasis. Further research in this direction will open up new avenues for novel GO applications in the prevention of cancer metastasis.

## 4. Materials and Methods

### 4.1. GO Preparation

GO was purchased from Nippon Shokubai Co, Osaka-Shi, Japan. Detailed characterization of GO was conducted via Fourier transform infrared spectroscopy, UV adsorption, and Raman spectra, and their results have been previously documented [46]. GO was suspended in pure water and adjusted to neutral pH, ranging from 7.0 to 7.5. Before treating cells, GO suspension was dispersed via sonication [58] using a microsonicator (UR-20P, Tomy Seiko, Tokyo, Japan) and diluted in cell culture medium that did not contain FBS.

### 4.2. Cell Culture

HCT-116 and HeLa cells were obtained from RIKEN BioResource Research Center (Wako, Japan). Cells were cultured in α-minimum essential medium (αMEM, FUJIFILM Wako Pure Chemical, Osaka, Japan) supplemented with 10% fetal bovine serum (Corning, New York, NY, USA), 100 µg/mL streptomycin, and 100 units/mL penicillin. Cells were grown under humidified conditions with 5% CO_2_ at 37 °C.

### 4.3. Treatment of Cells with GO

Non-adhered cells: Cells were gently detached from a culture dish via trypsin-EDTA treatment and suspended in αMEM containing 10% FBS. Detached cells were collected via centrifugation, and FBS-containing medium was completely removed. Cells were suspended in αMEM that lacked FBS and collected via centrifugation. Cells were resuspended in αMEM lacking FBS and treated with GO in the absence of FBS. After incubation with GO, FBS was added to the culture at 15%, and incubation was continued over the periods indicated in the figure legends. We note that the addition of FBS at 15% yielded more reproducible results than 10% FBS.

Adhered cells: Cells were cultured in αMEM containing FBS overnight to allow their adhesion. Medium was carefully removed, and adhered cells were washed twice with αMEM that lacked FBS. Adhered cells were treated with GO dispersed in αMEM that lacked FBS. After incubation with GO, FBS was added to the culture at 15% to quench the cytotoxic action of GO. We note that the addition of FBS at 15% yielded more reproducible results than 10% FBS. Incubation was continued over the periods indicated in the figure legends.

### 4.4. Measurement of Cell Viability and Intracellular ATP Level

Cell viability was measured using a Cell Counting Kit-8 (Dojindo Laboratories, Japan) and a microplate reader (MPR-A100, AsOne, Osaka, Japan). Cell lysis and ATP measurement were carried out using a CellTiter-Glo 3D cell viability assay reagent (G9682, Promega, Madison, WI, USA) according to the manufacturer’s instructions. Luminescence was measured using a 2030 ARVO X multilabel reader (Perkin Elmer, Shelton, CT, USA).

### 4.5. Fluorescence Microscopy

Cells in a glass-bottomed dish were stained with 1 µg/mL Hoechst 33342 (H342, Dojindo Laboratories, Kumamoto, Japan) and 0.25 µM MitoRed (R237, Dojindo Laboratories). A glass-bottomed dish was placed on a stage-top incubator (Tokai Hit, Fujinomiya, Japan) that maintained a humidified atmosphere and 5% CO_2_ at 37 °C. Fluorescence microscopy was carried out by using an FV1200-IX83 laser scanning confocal microscope with an oil-immersed 60× objective (Olympus, Tokyo, Japan). Hoechst 33342 was excited with 405 nm laser, and fluorescence at 460 nm was monitored. MitoRed was excited with 559 nm laser, and fluorescence at 580 nm was observed. Images were captured and analyzed using FLUOVIEW software (Version 4.1, Olympus, Tokyo, Japan).

### 4.6. Western Blot Analysis

Cells in a 35 mm dish were collected via scraping, followed by centrifugation. Cell pellets were washed with ice-cold phosphate-buffered saline and snap-frozen in liquid nitrogen. Frozen cell pellets were lysed in SDS-PAGE loading buffer containing 1% SDS. Cell lysates were sonicated and cleared via centrifugation at 20,000× *g* for 5 m. Proteins in the cleared lysates were resolved via SDS-PAGE, electrotransferred to a PVDF membrane, and reacted with primary antibodies according to standard procedures. Antigen-antibody complexes were detected via a chemiluminescence method using an HRP-conjugated secondary antibody and a Super Signal West Pico reagent (Thermo Fisher Scientific, Waltham, MA, USA). Chemiluminescence signals were detected using a ChemiDoc XRS Plus imaging system (Bio-Rad, Hercules, CA, USA) and quantified using ImageLab software (Version 2.0, Bio-Rad, Hercules, CA, USA). Antibodies used in this study were as follows:

rabbit anti-caspase 3 antibody (9662, Cell Signaling Technology, Danvers, MA, USA);
rabbit anti-LC3B antibody (ab192890, Abcam, Cambridge, UK);
mouse anti-β-actin antibody (A1978, Sigma-Aldrich, St. Louis, MO, USA);
anti-mouse HRP-linked IgG (7076S, Cell Signaling Technology, Danvers, MA, USA);
anti-rabbit HRP-linked IgG (7074S, Cell Signaling Technology, Danvers, MA, USA).

### 4.7. Statistical Analysis

Statistical analysis was carried out using Welch’s *t*-test. The number of experiments and *p*-values are indicated in the figure legends.

## Data Availability

The data that support the findings of this study are available from the corresponding authors upon reasonable request.
